# Audience Effects in Territorial Defense of Male Cichlid Fish Are Associated with Differential Patterns of Activation of the Brain Social Decision-Making Network

**DOI:** 10.3389/fnbeh.2017.00105

**Published:** 2017-05-31

**Authors:** António Roleira, Gonçalo A. Oliveira, João S. Lopes, Rui F. Oliveira

**Affiliations:** ^1^Instituto Superior de Psicologia Aplicada (ISPA)–Instituto UniversitárioLisbon, Portugal; ^2^Instituto Gulbenkian de CiênciaOeiras, Portugal; ^3^Champalimaud FoundationLisbon, Portugal

**Keywords:** audience effects, social decision-making network, immediate early genes, aggression, androgens, cortisol

## Abstract

Animals communicate by exchanging signals frequently in the proximity of other conspecifics that may detect and intercept signals not directed to them. There is evidence that the presence of these bystanders modulates the signaling behavior of interacting individuals, a phenomenon that has been named audience effect. Research on the audience effect has predominantly focused on its function rather than on its proximate mechanisms. Here, we have investigated the physiological and neuromolecular correlates of the audience effect in a cichlid fish (Mozambique tilapia, *Oreochromis mossambicus*). A male was exposed to a territorial intrusion in the presence or absence of a female audience. Results showed that the presence of the female audience increased territorial defense, but elicited a lower androgen and cortisol response to the territorial intrusion. Furthermore, analysis of the expression of immediate early genes, used as markers of neuronal activity, in brain areas belonging to the social decision-making network (SDMN) revealed different patterns of network activity and connectivity across the different social contexts (i.e., audience × intrusion). Overall, these results suggest that socially driven plasticity in the expression of territorial behavior is accommodated in the central nervous system by rapid changes in functional connectivity between nodes of relevant networks (SDMN) rather than by localized changes of activity in specific brain nuclei.

## Introduction

Group living animals establish communication networks in which individuals are within the signaling and receiving range of each other (McGregor, 2005). In such networks, social information may be broadcasted beyond the sender-receiver dyads toward unintended receivers. Therefore, it has been predicted that both senders and receivers may have co-evolved adaptations to take advantage of the social information available in communication networks, namely the evolution of eavesdropping in receivers and of changes in signaling behavior in senders in response to the presence of unintended receivers (aka audience effect; McGregor and Peake, 2000). Indeed, previous research has confirmed the occurrence of both types of adaptations in communicative behavior in different social species. For example, animals have been shown to use eavesdropping to monitor the aggressive interactions between conspecifics and the social information acquired is subsequently used to modulate their behavior in future interactions with the observed individuals (Oliveira et al., 1998). Moreover, there is evidence suggesting that the observation of agonistic interactions between third parties is sufficient to infer the social rank of conspecifics through transitive inference (Grosenick et al., 2007), and that eavesdropping itself is influenced by the observer's social status, with dominant eavesdroppers showing more interest in the losers than in the winners of an observed fight (Abril-de-Abreu et al., 2015). On the other hand, the presence of an audience has been shown to change mate choice (Plath et al., 2008) and food caching behavior (Leaver et al., 2007) of focal animals, in an attempt to conceal their decisions from the observing audience. Furthermore, the exposure to an audience before a fight has also been shown to prime the focal individual, which increases the expression of aggressive behavior toward its opponent hence reducing the time necessary to solve the agonistic interaction (Cruz and Oliveira, 2015).

Despite the numerous studies documenting the occurrence of social network phenomena, the proximate mechanisms (i.e., neural circuits, hormones, genes) involved in eavesdropping and the audience effect have been much less studied. It has been proposed that the adaptive behavioral responses to the social environment rely on the processing of social information by an evolutionary conserved network of multiple interconnected neural circuits, such that the overall activity across the network is expected to better reflect the behavioral output than the localized activity at a single node. This social decision-making network (SDMN; O'Connell and Hofmann, 2011, 2012) includes forebrain and midbrain structures that comprise the social behavior network (Newman, 1999; Goodson, 2005) and the mesolimbic reward system (Adinoff, 2004). The nodes of the social behavior network express receptors for hormones (sex steroids, glucocorticoids) and neuromodulators (e.g., oxytocin, vasopressin), suggesting that the state of the network can integrate information on the internal state of the animal (e.g., stress vs. not-stressed; breeding vs. non-breeding mode). Recent studies in zebrafish (*Danio rerio*) have shown that either gaining or losing social status induces the expression of different behavioral profiles which are paralleled by different patterns of immediate early gene expression across the SDMN and by different levels of brain nonapeptides and circulating sex steroids, hence supporting the hypothesis that different social behavior states rely on distributed processing of information across the SDMN paralleled by changes in central neuromodulatory state and in peripheral hormonal state (Teles et al., 2015, 2016; Teles and Oliveira, 2016). Thus, the effects of social context on signaling (i.e., audience effects) and bystander (i.e., eavesdropping) animals are predicted to rely, at the proximate level, on changes in the activity of SDMN paralleled by changes in internal state (i.e., hormonal and neuromodulatory state). Evidence for hormonal changes associated with audience and eavesdropping have both been documented. Experiments with male Siamese fighting fish (*Betta splendens*) have shown that androgen levels were lower when a female audience was present (Dzieweczynski et al., 2006), and higher levels of androgens were found in bystanders that were eavesdropping a fight between conspecifics in cichlid fish (Mozambique tilapia, *Oreochromis mossambicus*; Oliveira et al., 2001). The activity of the SDMN has also been studied in relation to social context in cichlid fish using the expression of immediate early genes as makers of neuronal activity. In a mate choice paradigm, *Astatotilapia burtoni* females showed a different pattern of activation of SDMN nodes after watching their preferred mate either winning or losing an agonistic interaction: SDMN nodes related to reproduction (i.e., pre-optic area and fish homolog of the ventromedial hypothalamus) were activated when their preferred mate won the fight, whereas nodes linked to anxiety (i.e., fish homolog of the lateral septum) were more active when their preferred mate lost the fight (Desjardins et al., 2010). In another study, relative body size has also been shown to influence the pattern of activation of SDMN nuclei, with larger body size inducing a higher activation of brain regions related to anxiety, both in fighting fish perceiving a audience made up of larger fish, or in eavesdroppers attending a fight between larger individuals (Desjardins et al., 2015). Finally, in zebrafish the brain transcriptomic changes associated with social eavesdropping have been shown to comprise an up-regulation of genes related to alertness and memory formation in fish eavesdropping a fight between conspecifics (Lopes et al., 2015). Together, these results suggest that the social context in which animals communicate changes the activity in relevant SDMN nuclei and the central and peripheral internal state of the interacting and eavesdropping animals, which is paralleled by changes in signaling behavior. However, the hypothesis that the processing of social information underlying audience and eavesdropping effects is encoded in the SDMN in a distributed fashion, rather than by the activity of a single node remains to be explicitly tested.

In this study, we tested the hypothesis that audience effects in contest behavior rely on integration of social information across nodes of the SDMN, rather than on regional specialization. For this purpose, we used Mozambique tilapia to test the effect of a female audience in the response of a territorial male to a territorial intrusion by a conspecific male. We hypothesized that the presence of the audience during territorial intrusions would induce different profiles of co-activation of the SDMN nodes, rather than localized differences in activity in specific nodes of the network. Neuronal activity was measured by the expression of two immediate early genes (*c-fos* and *egr-1*). Differences in neuroendocrine profiles were also investigated using androgen (i.e., 11-ketotestosterone, KT and testosterone, T) and cortisol (F) levels as indicators of the activity of the hypothalamic-pituitary-gonadal (HPG) and hypothalamic-pituitary-interenal (HPI) axes, respectively.

## Materials and methods

### Ethics statement

All procedures were conducted in accordance with the European Communities Council Directive of 24 November 1986 (86/609/EEC) for the care and use of animals in experimental procedures, and were approved by the Portuguese Veterinary Authorities (Direcção Geral de Alimentação e Veterinária, Portugal; permit # 0421/000/000/2013).

### Experimental animals and housing conditions

Forty-eight *O. mossambicus* males (mean ± standard deviation: length, 10.5 ± 0.65 cm; body mass, 36.18 ± 6.67 g) from a stock maintained at ISPA-IU (Lisbon, Portugal) were used in this study. Only males that were territorial (as characterized according to Oliveira, 1995) in the stock tanks were selected as focal animals. These males were housed together with females (8 males and 6 females per 160 L tank) in aquaria with fine gravel substrate, a double filtering system (both sand and external biofilter, TETRA) and constant aeration. These aquaria were kept at 26 ± 2°C, with a photoperiod of 12 h light: 12 h dark. Fish were daily fed *ad libitum* with commercial cichlid sticks (TETRA). Water quality was monitored once a week with Pallintest kit® for nitrites (0.001–0.5 ppm) and ammonia (<0.5 ppm).

### Experimental procedures

**Figure 1:**
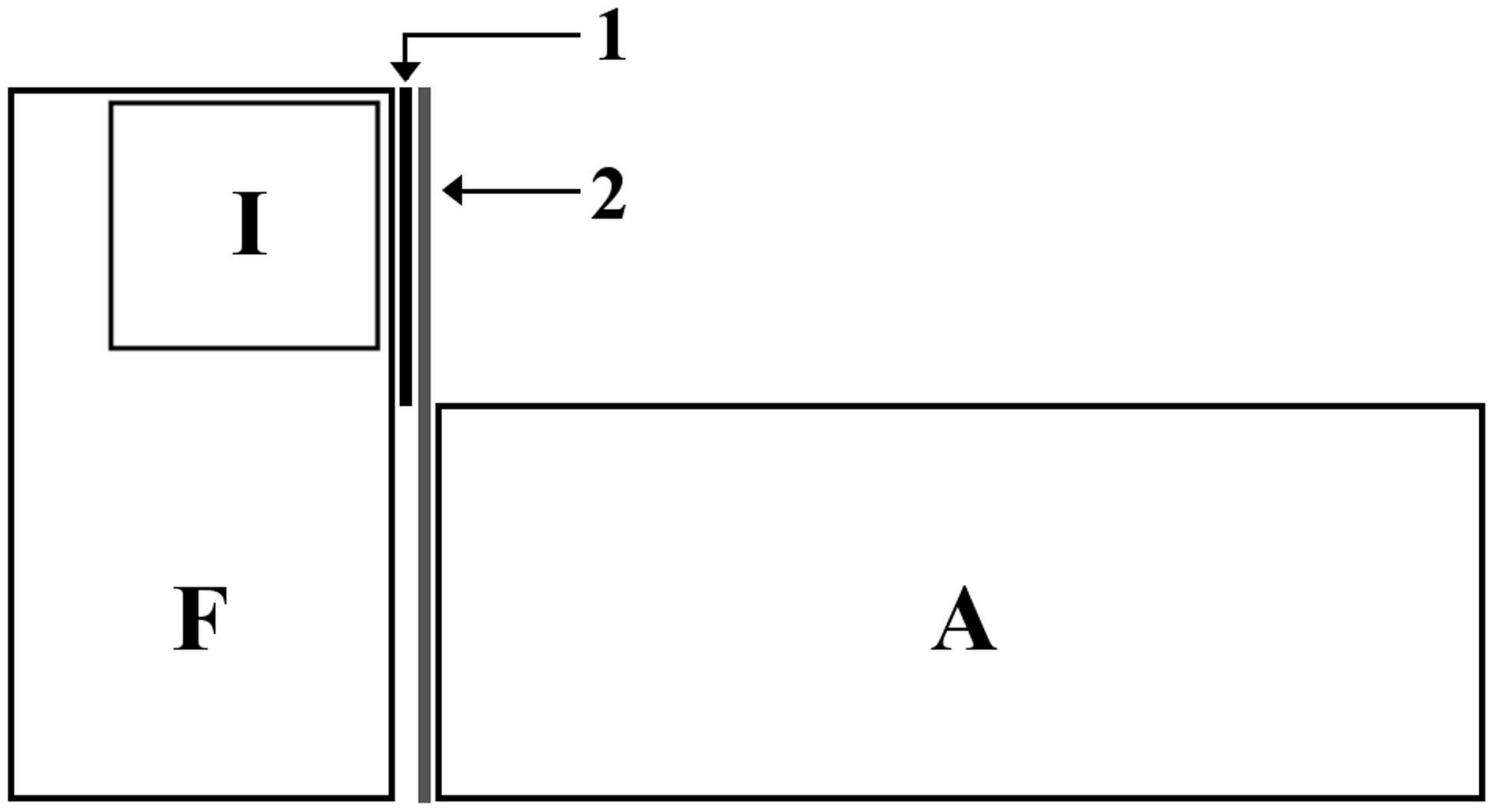
Experimental set up: F, Focal tank (glass); I, Intruder tank (glass; placed inside the focal tank) with holes to allow chemical communication; A, Audience tank (glass); 1, permanent barrier to prevent visual communication between intruders and audience; 2, Unidirectional mirror to prevent audience to see and interact with focal males.

Focal males were placed individually in the experimental tanks (30 L) 48 h before the test for acclimatization. Inside each experimental tank there was a compartment made of non-sealed transparent walls, where the intruder could be introduced. Thus, focal males had visual and chemical access to the intruders. During the acclimatization phase, focal males had visual (but not chemical) access to an audience tank composed entirely by females. In order to prevent interactions between the audience and the focal fish, a one-way mirror was placed between the experimental and the audience tank, such that focal males were aware of the presence of the audience but the audience could not see the focal fish (Figure 1).

All focal males established territories, built nests, and adopted the nuptial coloration in the experimental tanks during the 48 h acclimatization phase. Females were chosen as an audience to promote a mating social context, since territorial intrusions occur in breeding territories and a previous study showed that socially isolated males were highly motivated to come in close contact with females (Galhardo et al., 2011). Only unfamiliar females were used as an audience to prevent any effects of previous experience.

Four experimental treatments (see Table S2 in the Supplementary Material for detailed sample sizes by treatment and variable) were conducted manipulating territorial intrusions and the presence of an audience in a 2 × 2 design: (i) Intrusion with an Audience (I.A.)—focal male faced a territorial intrusion in the presence of a female audience; (ii) Intrusion with No Audience (I.NA.)—focal male faced a territorial intrusion in the absence of a female audience; (iii) No intrusion but with an Audience (NI.A.)—focal male had visual access to the female audience, but did not face a territorial intrusion; and (iv) No Intrusion and No Audience (NI.NA.)—focal male in social isolation.

Trials started between 8:30 a.m. and 9:30 a.m. and lasted for 30 min. They were video recorded, starting 5 min before the experiment using a video camera placed at the opposite side of the audience tank, hence allowing for the visualization of the three tanks. In the treatments without audience, immediately after starting the camera, an opaque partition was placed between the one-way mirror and the audience aquarium. In the territorial intrusion treatments, the intruder male was placed in the intruder's tank after turning on the camera and the experiment started immediately after the entrance of the intruder. In order to standardize the threat posed by intruders toward resident males, all intruders were dominant males in their home tanks and were size matched with the focal fish [mean ± standard deviation: Length – Focal: 10.50 ± 0.13 cm, Intruder: 10.59 ± 0.11 cm, *t*_(23)_ = 0.64, *p* = 0.53; Body mass – Focal: 35.25 ± 1.23 g, Intruder: 36.39 ± 1.06 g, *t*_(23)_ = 0.79, *p* = 0.44].

### Behavioral observations

For behavioral quantification, 30 min video recordings were analyzed using a multi-event recorder software (The Observer XT 7.0, Noldus, Wageningen, the Netherlands). The frequency and duration of the following behavioral patterns were quantified: bites, displays and interactions with the glass partition (i.e., attempts to interact with the audience by swimming against the glass, but without any attempts to attack; for an ethogram of this species, see Baerends and Baerends-Van Roon, 1950).

### Hormone assays

Immediately after the behavioral trial, the focal animal was quickly anesthetized (MS-222, Pharmaq; 300 ppm) and blood samples were taken from the caudal vein (using 1 ml syringes with 25 G/16 mm needles). Blood sampling always took <4 min to avoid effects of handling stress on sampled F levels (Foo and Lam, 1993). Blood samples were centrifuged at 3000G for 10 min at 4°C, and the plasma was collected and stored at −20°C until further processing. The free hormone fraction was extracted from the plasma by adding 2 ml of diethyl ether (#1.00921.1000, Merck) to each sample, and stirring for 20 min. Samples were then centrifuged at 93 G, for 5 min at 4°C. The ether fraction was then separated by freezing the samples twice (10/15 min, −80°C). The steroids were isolated by evaporating the ether in Speed-Vac (Savant instruments) for 20 min. The dried organic phase containing the free steroid fraction was then reconstituted in 1 ml phosphate buffer solution. Samples were stored at −20°C until the hormone assay. Levels of T, KT, and F were quantified by radioimmunoassay as described by Galhardo and Oliveira (2013) (see Supplementary Information for details on antibodies and radioactive hormone markers used). All samples were analyzed within the same assay and the intra-assay variability were 0.75% for F; 2.68% for testosterone; and 3.74% for KT.

### Microdissection of the regions of interest in the SDM network

After blood sampling, fish were deeply anesthetized and hen sacrificed by decapitation. Fish heads were collected and embedded in Tissue-Tek^β^ (OCT—optimal cutting temperature compound, Sakura), frozen at −80°C, and sectioned in the coronal plane into 300 μm thick sections using a cryostat (Microm HM500 M, Waldorf—Germany) and mounted on glass microscope slides. The microdissections were performed under a stereoscope (Stereomicroscope VWR SZB350OH) with the slides placed on a cold plate using a modified 25G needle attached to a syringe. The collected tissue was pooled directly into a 1.5 ml Eppendorf with 50 μl of QIAzol^β^ Lysis Reagent (RNeasy Lipid Tissue Mini Kit, Qiagen) and stored at −80°C until mRNA extraction. To prevent cross contamination between samples, each needle was only used to sample the same brain nuclei and the needles were cleaned sequentially with distilled water and ethanol 70% between the sampling of different individuals. Regions of interest in the SDM network (O'Connell and Hofmann, 2011) were identified using the *O. mossambicus* brain atlas (Simões et al., 2012). The following nuclei were collected in both hemispheres: dorsomedial telencephalon (Dm, putative homolog of the basolateral amygdala in mammals), dorsolateral telencephalon (Dl, putative homolog of mammalian hippocampus), ventral subdivision of the ventral telencephalon (Vv, putative homolog of the mammalian lateral septum), supracommissural part of the ventral telencephalon (Vs, putative homolog of the mammalian medial extended amygdala and the bed nucleus of the stria terminalis), preoptic area (POA), nucleus anterior tuberis (TA, putative homolog of mammals ventromedial hypothalamus), and central gray (GC).

### Gene expression analysis

Total RNA was isolated from brain nuclei using the RNeasy Lipid Tissue Mini Kit with some adjustments to the manufacturer's instructions (see the electronic Supplementary Material for details). RNA from each sample was then reverse transcribed to cDNA with iScript cDNA Synthesis Kit (Biorad) in accordance with manufacturer's instructions and diluted 1:10 before being used as a template for quantitative polymerase chain reactions (RT-PCR) of *c-fos* and *egr*-1, using the eukaryotic translation elongation factor 1 alpha (*elf1a*) as a reference gene (see the electronic supplementary material for details, especially Table S1 for primer sequences). Fluorescence cycle thresholds (CT) were automatically measured (Biosystems 7900HT Fast thermocycler) and the relative expression of the target genes was calculated using the 2^−ΔCt^ method. This protocol was conducted based on previous research developed in our lab (Teles et al., 2015).

### Statistical analysis

In order to match parametric test assumptions, a logarithmic transformation was applied to F levels [log_10_(x)], gene expression ([log_10_(x)] and behavioral variables [log_10_(x+1)]. Outlier observations were identified using a generalized extreme studentized deviate procedure (*p* = 0.05, maximum number of outliers set to 20% of the sample size) and removed from the samples (see Table S3 in the Supplementary Material for details). Differences in aggressive behavior toward the intruder between the I.A and I.NA treatments were assessed using *t*-tests. Separate Linear Mixed Models with the individual focal males as a random effect and Intrusion (No intrusion, Intrusion) and Audience (No audience, Audience) as fixed effects were used to test for overall differences (i.e., main effects of each factor and interaction between the two factors) in each dependent variable, namely: frequency and duration of interactions with the audience, levels of T, F, and 11KT. IEG expression in the brain was also tested using separate Linear Mixed Models with *c-fos* or *egr-1* expression in each brain area (Dm, Dl, Vv, Vs, POA, TA, GC) nested within a fixed effect, Intrusion (No intrusion, Intrusion) and Audience (No audience, Audience) also as fixed effects and finally, individual focal fish was inserted as a random effect. In all models, planned comparisons (*z*-tests within the Linear Mixed Models) were defined a priori to test for the specific effects of the presence of the audience (NI.A vs. NI.NA; I.A vs. I.NA) and of the territorial intrusion (I.A vs. NI.A; I.NA vs. NI.NA).

To test for functional connectivity, Pearson correlation matrices of each pair of brain nuclei for each of the measured IEGs were tested using the Quadratic Assignment Procedure (QAP) with 5,000 permutations. In line with the QAP null-hypothesis, a non-significant result indicates that there is no association between the IEG activational pattern of the experimental conditions being tested. SDM networks in each treatment were characterized using eigenvector as a measure of centrality and density as a measure of network cohesion. Differences in network density between conditions were tested using a *t*-test bootstrapped to 5,000 sub-samples. *P*-values were adjusted for the number of tests using the Benjamini and Hochberg (1995) correction for planned comparisons in the Linear Mixed Models and for the Pearson correlations. Statistical analyses were performed using the following R packages (R Core Team, 2015): nlme (linear mixed models), multcomp (planned comparisons), Hmisc (correlations), and ggplots (heatmaps). Characterization of the SDM network was performed using UCINET v.6. Degrees of freedom vary between the analyses due to missing values in the raw data or as result of the outlier detection procedure.

## Results

### Behavior toward the intruder

**Figure 2:**
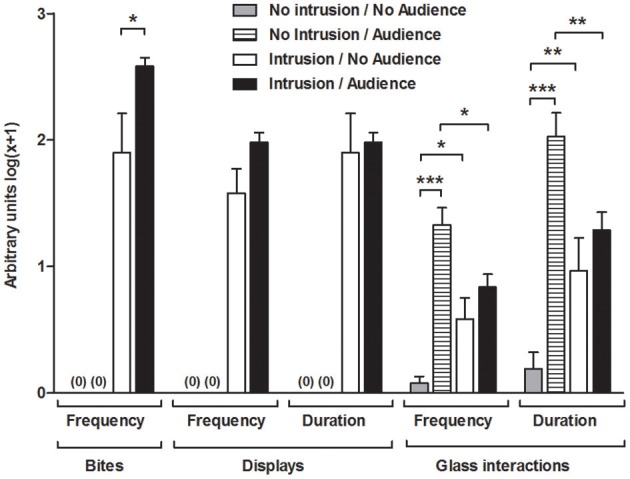
Behavioral measurements for the focal fish in each experimental condition. ^*^Significant difference for *p* < 0.05; ^**^significant difference for *p* < 0.01; ^***^Significant difference for *p* < 0.001.

The territorial intrusions in the presence of the audience were characterized by a significantly higher frequency of bites compared to the intrusions without an audience [I.A vs. I.NA: *t*_(22)_ = 2.171, *p* = 0.041], but the audience had no significant effect on the frequency [I.A vs. I.NA: *t*_(22)_ = 1.955, *p* = 0.063] or duration of displays [I.A vs. I.NA: *t*_(22)_ = 1.109, *p* = 0.279] (Figure 2).

### Behavior toward the audience

Overall, the presence of the audience increased the frequency [*F*_(1, 42)_ = 32.504, *p* < 0.001] and duration [*F*_(1, 42)_ = 29.803, *p* < 0.001; Figure 2] of the behaviors toward the glass partition and these behavioral parameters were modulated by the territorial intrusion [Frequency, Intrusion × Audience: *F*_(1, 42)_ = 15.107, *p* < 0.001; Duration, Intrusion × Audience: *F*_(1, 42)_ = 15.583, *p* < 0.001]. The planned comparisons showed that the territorial intrusion decreased the behaviors directed toward the audience (I.A vs. NI.A, frequency: *z* = 2.769, *p* = 0.008; duration: *z* = 2.797, *p* = 0.007), and the behavior toward the audience during a territorial intrusion was not significantly different from that of the treatment with an intrusion in the absence of an audience (I.A vs. I.NA, frequency: *z* = 1.436, *p* = 0.151; duration: *z* = 1.220, *p* = 0.222). Comparisons with fish in social isolation showed a higher number and duration of behaviors on the glass partition when the audience was present (NI.A vs. NI.NA, frequency: *z* = 6.739, *p* < 0.001; duration: *z* = 6.617, *p* < 0.001) and when there was a territorial intrusion in the absence of the audience (I.NA vs. NI.NA, frequency: *z* = 2.730, *p* = 0.008; duration: *z* = 2.787, *p* = 0.007).

### Hormone levels

**Figure 3:**
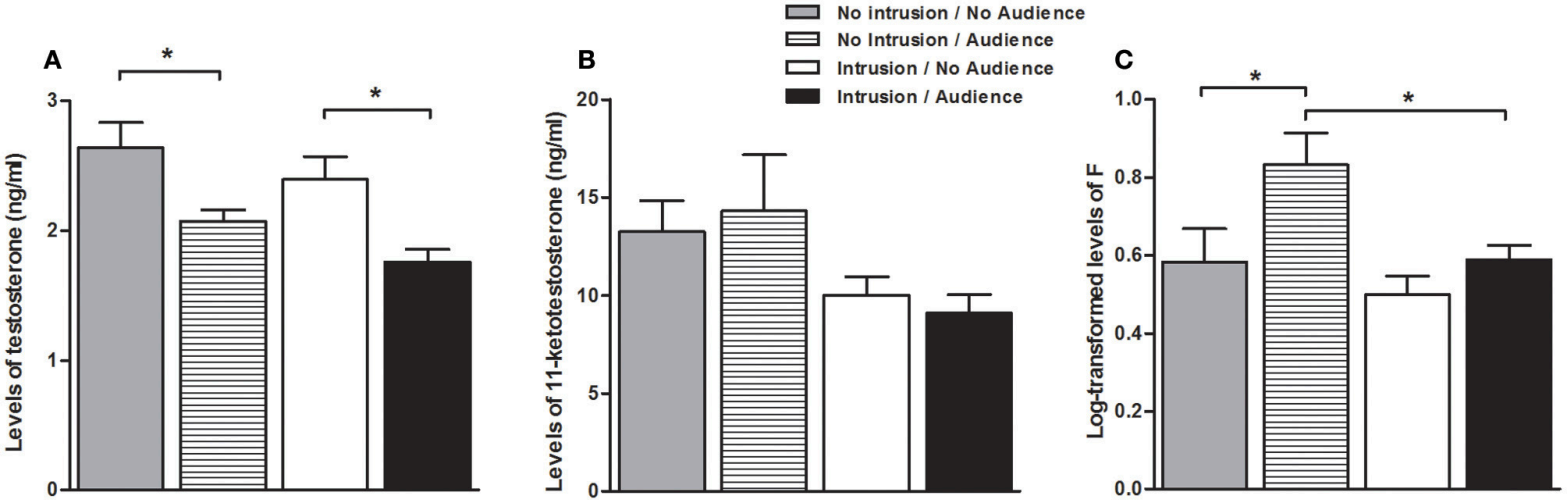
Hormone levels for the focal fish in each experimental condition. **(A)** Testosterone levels; **(B)** 11-Ketotestosterone levels; **(C)** Cortisol levels; ^*^significant difference for *p* < 0.05.

There was a main effect of the presence of the audience both on T levels [higher when the audience was absent; *F*_(1, 40)_ = 15.806, *p* < 0.001], and on F levels [higher when the audience was present; *F*_(1, 42)_ = 6.324, *p* = 0.016], whereas no main effect of the audience was detected on KT levels [*F*_(1, 40)_ < 0.001, *p* = 979; Figure 3].There was also a main effect of the territorial intrusion both on KT and F levels [lower with territorial intrusion, in both cases; KT: *F*_(1, 40)_ = 5.031, *p* = 0.030; F: *F*_(1, 42)_ = 5.697, *p* = 0.022], while it had no main effect on T levels[*F*_(1, 40)_ = 3.344, *p* = 0.075; Figure 3]. There were no significant interaction effect between audience and territorial intrusion in the levels of any of the hormones [T: *F*_(1, 40)_ = 0.049, *p* = 0.826; KT: *F*_(1, 40)_ = 0.276, *p* = 0.602; F: *F*_(1, 42)_ = 1.350, *p* = 0.252; Figure 3].

Planned comparisons (see Table 1 for all planned comparisons) showed that T levels were lower when an audience was present irrespective of a territorial intrusion (I.NA vs. I.A; NI.A vs. NI.NA). In contrast, F levels were higher when an audience was present in the absence of a territorial intrusion but not when exposed to a territorial intrusion (NI.A vs. NI.NA). F levels also showed a response to the territorial intrusion when the audience was present (NI.A vs. I.A), with fish exposed to the intrusion displaying lower F levels. Planned comparisons did not detect any significant difference between the specific treatments for KT levels.

**Table 1:** Planned comparisons between conditions for hormone levels and immediate early gene expression in the brain.

	**Measure**	**Intrusion/Audience vs. Intrusion/No Audience**		**Intrusion/Audience vs. No Intrusion/Audience**		**Intrusion/No Audience vs. No Intrusion/No Audience**		**No Intrusion/Audience vs. No Intrusion/No Audience**	
		***z***	***p***	***z***	***p***	***z***	***p***	***z***	***p***
*Hormones*	T	2.909	0.013^*^	1.423	0.206	1.169	0.242	2.724	0.013^*^
	11KT	0.332	0.740	1.990	0.186	1.160	0.492	0.417	0.740
	F	0.900	0.386	2.509	0.024^*^	0.866	0.386	2.620	0.024^*^
*c-fos*	Dm	0.541	0.867	1.309	0.590	0.957	0.729	1.704	0.412
	Dl	0.812	0.777	1.614	0.426	0.544	0.867	0.262	0.965
	Vv	0.192	0.981	0.551	0.867	0.109	0.981	0.825	0.777
	Vs	0.397	0.938	1.455	0.510	1.068	0.723	0.023	0.981
	POA	0.380	0.938	0.033	0.981	0.653	0.867	1.015	0.723
	TA	1.903	0.319	1.224	0.618	2.904	0.059	2.198	0.195
	GC	0.304	0.966	0.090	0.981	2.732	0.059	2.751	0.059
*egr-1*	Dm	0.868	0.831	1.441	0.697	2.329	0.273	1.781	0.419
	Dl	0.494	0.831	0.706	0.831	0.797	0.831	2.120	0.273
	Vv	0.646	0.831	2.063	0.273	4.104	0.001^**^	0.921	0.831
	Vs	0.424	0.831	0.009	0.993	0.569	0.831	0.972	0.831
	POA	0.207	0.900	0.520	0.831	0.086	0.966	0.607	0.831
	TA	0.368	0.831	0.378	0.831	0.222	0.900	1.018	0.831
	GC	0.802	0.831	0.395	0.831	0.559	0.831	0.617	0.831

*T, Testosterone; 11KT, 11-ketotestosterone; F, Cortisol; Dm, dorsomedial telencephalon; Dl, dorsolateral telencephalon; Vv, ventral telencephalon; Vs, supracommissural nucleus of the ventral telencephalon; POA, preoptic area; TA, nucleus anterior tuberis; GC, central gray*.

* Significant difference for p < 0.05;

** *Significant difference for p < 0.01*.

### Activation of nodes of the social decision-making network

**Figure 4:**
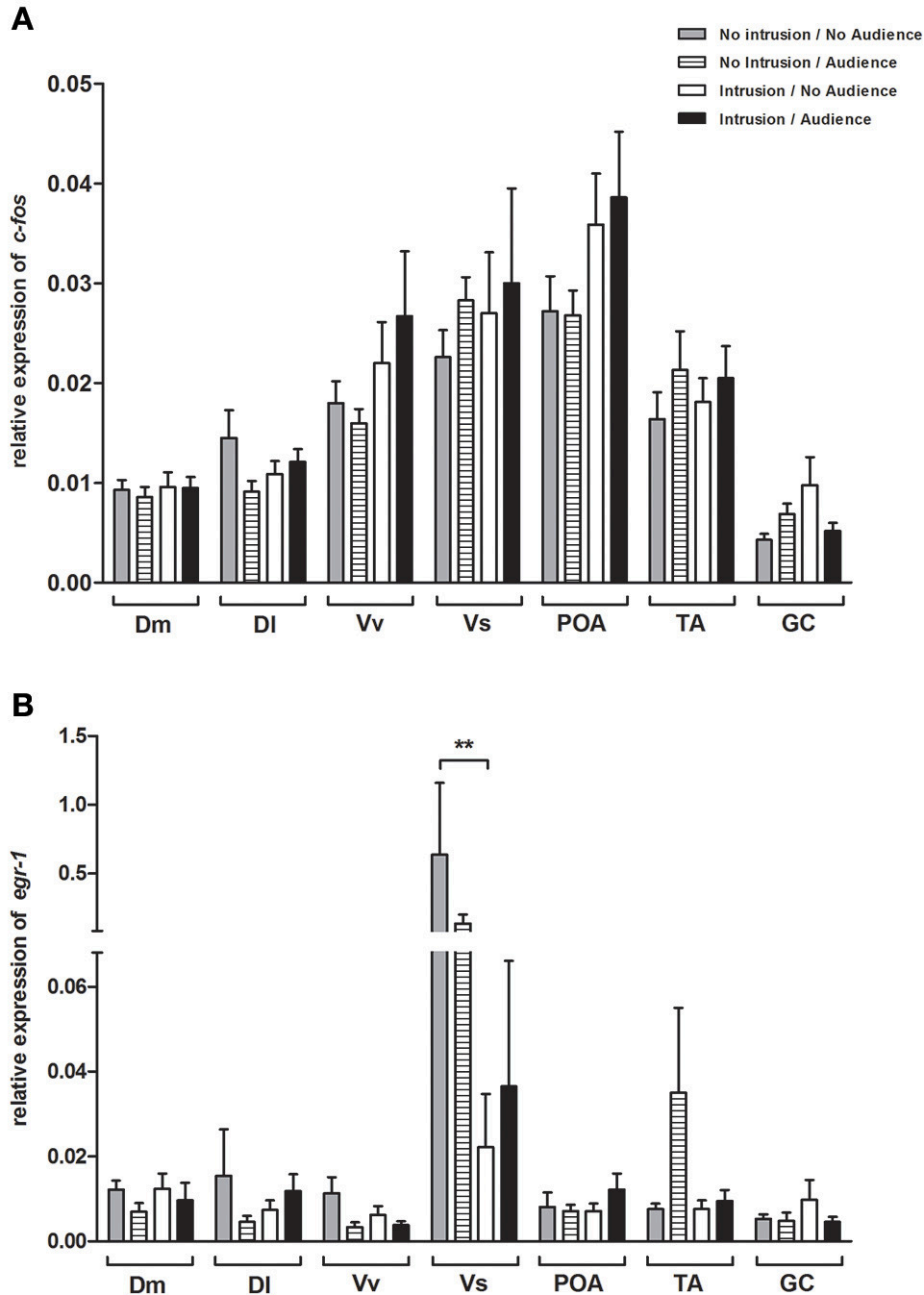
Expression of immediate early genes in brain areas of the SDM network (A) *c-fos*; (B) and *egr-1*. ^*^Significant difference for *p* < 0.05; ^**^Significant difference for *p* < 0.01.

No main effect or interaction for territorial intrusions or for audience was detected for *c-fos* [Intrusion: *F*_(1, 35)_ = 2.406, *p* = 0.129; Audience: *F*_(1, 35)_ = 0.203, *p* = 0.655; Intrusion × Audience: *F*_(1, 35)_ = 0.047, *p* = 0.829; Figure 4A] or for *egr-1* [Intrusion: *F*_(1, 44)_ = 0.331, *p* = 0.567; Audience: *F*_(1, 44)_ = 0.055, *p* = 0.815; Intrusion × Audience: *F*_(1, 44)_ = 0.131, *p* = 0.718; Figure 4B]. The planned comparisons between treatments only revealed a significant difference for the expression of *egr-1* in the Vs (see Table 1, Figure 4B), with fish in social isolation (NI.NA) showing higher expression of *egr-1* than those in that received an intrusion without the presence of an audience (I.NA).

### Functional connectivity across the social decision-making network

**Figure 5:**
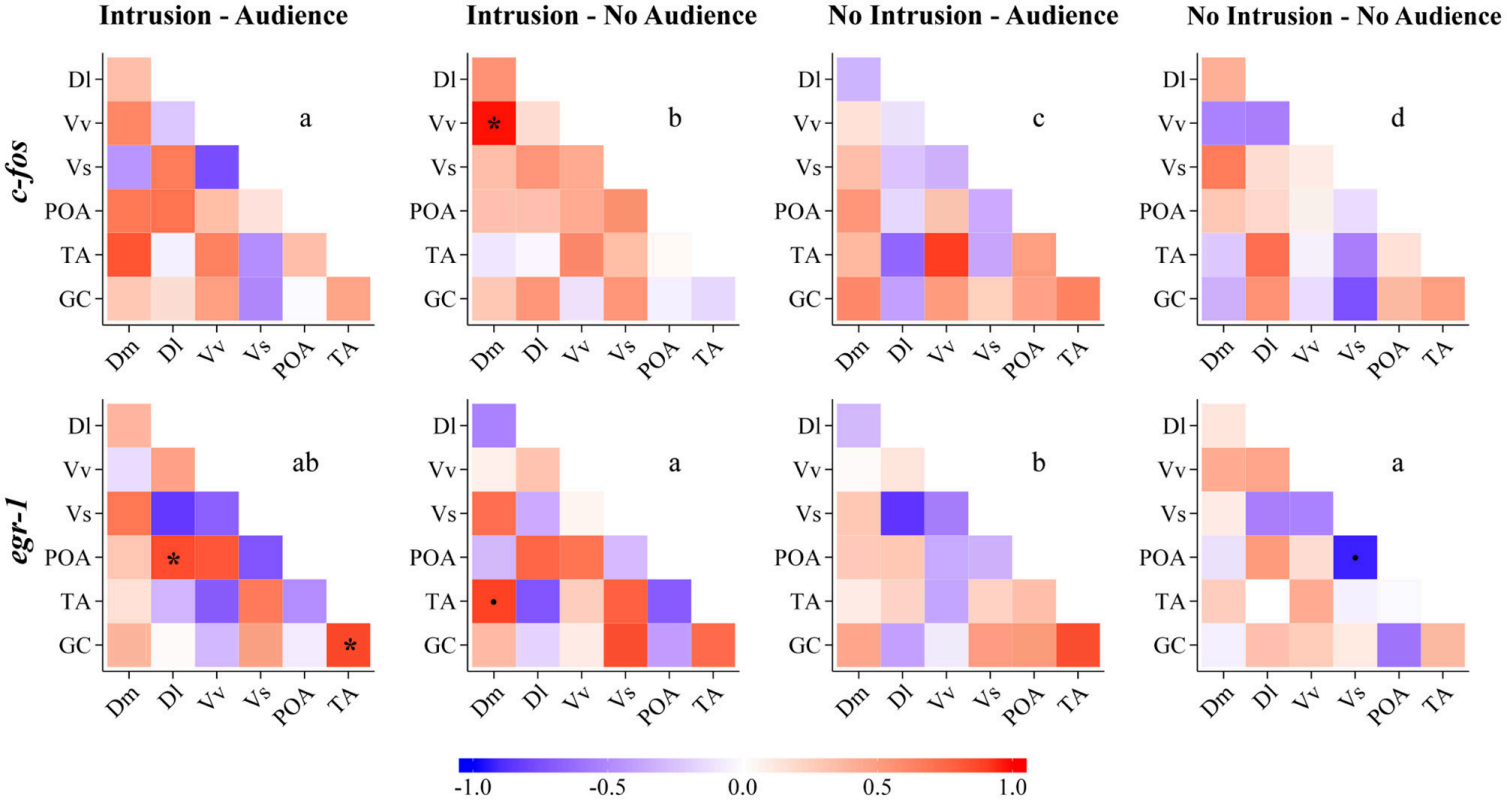
Functional connectivity in the SDM network for all the experimental treatments as measured by Pearson correlations between pairs of brain nuclei for *c-fos* and *egr-1*. Dm, medial zone of the dorsal telencephalic area; Dl, lateral zone of the dorsal telencephalic area; Vv, ventral nucleus of the ventral telencephalic area; Vs, supracommissural nucleus of the ventral telencephalic area; POA, preoptic area; TA, nucleus anterior tuberis; GC, central gray. ^*^Significant correlation for *p* < 0.05; (.) marginal correlation *p* < 0.10. Different letters indicate significantly different patterns of IEG expression in brain nuclei between treatments using the QAP correlation test.

The comparisons of the correlation matrices for the expression of *c-fos* across the nodes of the SDMN using QAP showed that all treatments had a distinct co-activation pattern (Figure 5, Table 2). On the other hand, QAP tests showed that the expression patterns of *egr-1* were only significantly different between the NI.A treatment and both the I.NA and NI.NA treatments. All the other comparisons showed a similar co-activation pattern for *egr-1* (Figure 5, Table 2).

**Table 2:** Quadratic assignment procedure results for *c-fos* and *egr-1* co-activation matrices in the brain.

		**I.A**		**NI.A**		**I.NA**	
		***r***	***p***	***r***	***p***	***r***	***p***
*c-fos*	NI.NA	0.112	0.283	−0.110	0.329	−0.286	0.101
	I.NA	−0.033	0.461	−0.256	0.163		
	NI.A	0.370	0.105				
*egr-1*	NI.NA	0.518	0.014	0.260	0.151	0.447	0.028
	I.NA	0.650	0.025	0.295	0.097		
	NI.A	0.579	0.003				

*I.A., Intrusion with an Audience; I.NA., Intrusion with No Audience; NI.A., No intrusion but with an Audience; NI.NA., No Intrusion and No Audience*.

Regarding the network density, no significant differences were detected between treatments for *c-fos* (I.A vs. NI.A: *t* = 0.617, *p* = 0.517; I.A vs. I.NA: *t* = 1.837, *p* = 0.067; I.NA vs. NI.NA: *t* = 0.018, *p* = 0.982; NI.A vs. NI.NA: *t* = 0.833, *p* = 0.379), whereas for *egr-1* expression the I.A treatment had a significantly higher network density than the NI.A treatment using *egr-1* expression (I.A vs. NI.A: *t* = 4.121, *p* = 0.001). No other differences were found between the conditions for *egr-1* network density (I.A vs. I.NA: *t* = 0.176, *p* = 0.868; I.NA vs. NI.NA: *t* = 1.856, *p* = 0.062; NI.A vs. NI.NA: *t* = 1.258, *p* = 0.193).

**Figure 6:**
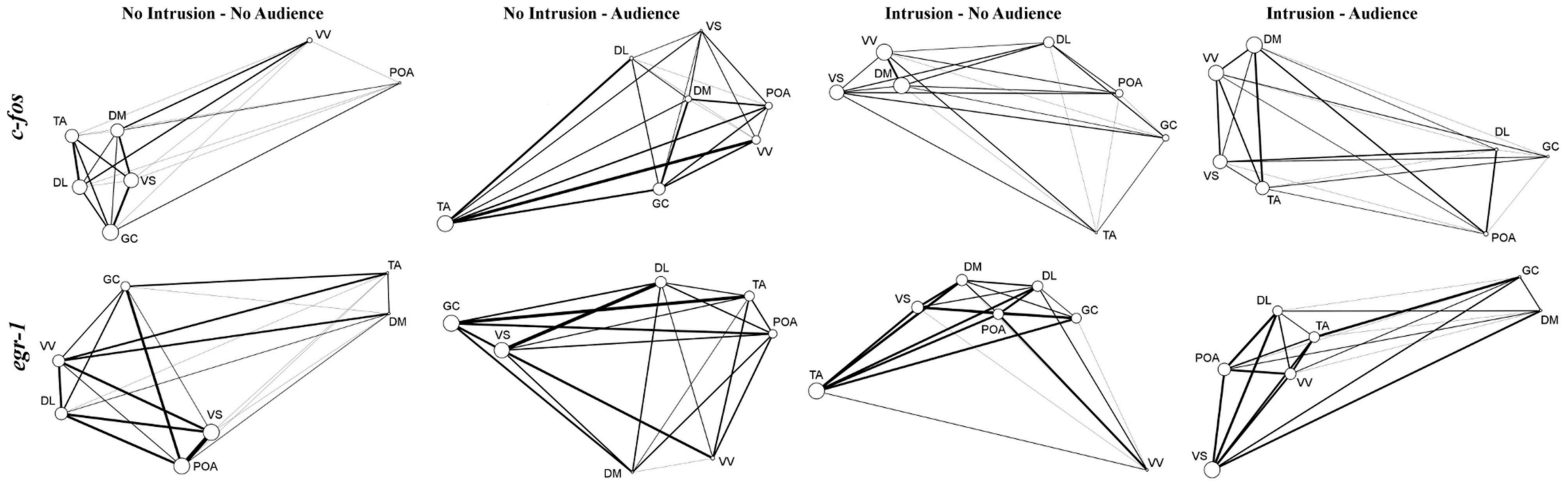
Representations of the SDM network for all the experimental treatments clustered by eigenvector centrality treatment and using *c-fos* and *egr-1* as reporters of neuronal activity. Node size indicates eigenvector centrality. Line size indicates the strength of the connection between nodes.

Regarding network centrality, in the *c-fos* network the most well connected nodes in the treatments with territorial intrusions (I.A and I.NA) were Dm, Vv, and Vs, while in the treatment without territorial intrusion but with an audience (NI.A) the TA and GC are the nodes receiving more connections (Table 3, Figure 6). When the focal fish is in social isolation (NI.NA) several areas present similar eigenvector scores for centrality, with Vv and POA showing the least amount of connections. Measures of centrality for the *egr-1* network showed that Vs is a common important node in all treatments (Table 3, Figure 6), while other nodes seem to be more central in each of the other treatments (POA for NI.NA and I.A, GC for NI.A, and TA for I.NA).

**Table 3:** Characterization of the SDM network for each experimental treatment using *c-fos* and *egr-1* as reporters of neuronal activity.

		***c-fos***				***egr-1***			
		**NI.NA**	**NI.A**	**I.NA**	**I.A**	**NI.NA**	**NI.A**	**I.NA**	**I.A**
*Density*		0.351	0.401	0.352	0.434	0.302	0.352	0.476	0.490
*Eigenvector*	Dm	0.400	0.347	0.453	0.440	0.198	0.265	0.389	0.261
	Dl	0.426	0.312	0.374	0.304	0.422	0.395	0.370	0.381
	Vv	0.262	0.381	0.456	0.433	0.397	0.281	0.191	0.402
	Vs	0.423	0.291	0.439	0.417	0.486	0.455	0.401	0.477
	POA	0.221	0.359	0.328	0.317	0.491	0.360	0.377	0.419
	TA	0.400	0.491	0.238	0.408	0.191	0.374	0.497	0.389
	GC	0.449	0.428	0.301	0.293	0.334	0.466	0.355	0.266

*Values reported correspond to network cohesion (density) and centrality (eigenvector) of each node of the network*.

*Dm, Dorsomedial telencephalon; Dl, dorsolateral telencephalon; Vv, ventral telencephalon; Vs, supracommissural nucleus of the ventral telencephalon; POA, preoptic area; TA, nucleus anterior tuberis; GC, central gray; I.A., Intrusion with an Audience; I.NA., Intrusion with No Audience; NI.A., No intrusion but with an Audience; NI.NA., No Intrusion and No Audience*.

## Discussion

### Audience effects on territory defense

The behavioral analysis showed that the conceived procedure created successfully the experimental treatments intended by our experimental design. In fact, focal males showed interest in the female audience when it was available, especially in the treatment with no territorial intrusion. In this condition, males had longer and more frequent interaction attempts with the females, matching previous research showing that females are a valuable resource for males and that males of this species are highly motivated to get access to them (Galhardo et al., 2011). The presence of a female audience also modulated the aggressive behavior of the focal males, by increasing the frequency of bite attacks toward territorial intruders. This increased aggressive response toward intruders goes in the same direction of that reported for another cichlid fish (*Astatotilapia burtoni*; Desjardins et al., 2010), in which focal males were more aggressive in the presence of a male audience. Interestingly, experiments with Siamese fighting fish (*Betta splendens*) showed that aggression in agonistic interactions also increases in the presence of a male audience, but decreases in the presence of a female audience (Matos and McGregor, 2002; Dzieweczynski et al., 2005). These findings for the female audience have been interpreted as a trade-off between confronting the intruder and not driving away the females (Matos and McGregor, 2002). In Mozambique tilapia males form dense breeding aggregations in shallow waters, where dominant males establish individual territories centered in a spawning pit (aka bower) that they mouth-dig, and floater males move around trying to take-over territory holders (Baerends and Baerends-Van Roon, 1950; Turner, 1986; Oliveira and Almada, 1998). Females visit these male breeding aggregations when ready to spawn and select a mate based on bower size (Mindy Nelson, 1995). Females collect and mouth-brood the eggs after spawning in separate nursery areas, hence the bower is only used as a male display and as a spawning surface. Thus, the cost of losing a territory (with a bower) to an intruder is higher when females are present since it represents a lost mating opportunity. This may explain our results, with higher escalation by the territory owner in the presence of the female audience.

### Audience effects on hormonal state

Although previous research in Siamese fighting fish found lower KT levels in response to territorial intrusions when a female audience is present (Dzieweczynski et al., 2006), we did not detect any effect of the presence of the female audience in KT levels. However, our results for T, another androgen that has been associated with the expression of aggressive behavior in tilapia (Oliveira et al., 1996), are congruent with the findings for Siamese fighting fish, since our focal males that faced a territorial intrusion had lower T levels when the female audience was present. Thus, in our study the presence of a female audience during a territorial intrusion decreased T levels of territorial males, while paradoxically increasing their levels of aggression (e.g., Matos and McGregor, 2002).

A recent experiment with convict cichlids (*Amatitlania nigrofasciata*; Scarsella et al., 2016), in which animals had an agonistic encounter across a clear partition, found a decrease in T and F levels in response to the interaction. This result is in line with previous research in tilapia that had shown that unresolved interactions fail to trigger an androgen response (Oliveira et al., 2005). These studies may explain why T levels were lower for intrusions in the presence of an audience, since in our experimental setup the territorial male also interacted with the intruder across a clear partition, and therefore no definite resolution of the social challenge could be achieved. Furthermore, the lower F levels in the treatment with the territorial intrusion in the presence of an audience, when compared to focal males that had an audience present but were not challenged by an intruder also corroborates this interpretation and replicates the findings of Scarsella et al. (2016). It is also reasonable to assume that in our experiment the effects of the female audience and of the inability to resolve the agonistic interaction on lowering the T levels were cumulative, since the comparison between the treatments without territorial intrusions also showed that T levels were lower when the female audience was present.

In our study, we have also found that F levels were higher in males that had an audience present but no territorial intrusion than in males in social isolation. This result contrasts with previous work on this species that has shown that social isolation is a major stressor that induces a significant increase in F levels (Galhardo and Oliveira, 2013). One possible explanation for this contradictory result can be the fact that we have used a one-way mirror to separate the focal male from the female audience, hence allowing the male to see the audience, but preventing the audience from seeing and interacting with the focal male. As a consequence, the female audience was unresponsive to the male attempts to interact with it, and this lack of response may have been perceived as a social stressor by the focal male, hence triggering an increase in F levels.

### Audience effects on the brain social-decision making network

We have used the expression of two commonly used immediate early genes, *c-fos* and *egr-1*, as markers of neuronal activity. Interestingly, the patterns of brain activity reported by each of these genes are not coincident. Such discrepancies have been reported in other studies that used both genes as reporters of neuronal activity (Desjardins et al., 2010; Teles et al., 2015), and maybe due to the fact that although both are activated by neuronal activity they are involved in different signaling pathways that may have different dynamics. Therefore, an integrated analysis of the expression of both immediate early genes should be used when trying to infer patterns of brain activity from their expression.

In this experiment, we have not found significant differences in the activity of a single node of the SDMN that allowed us to distinguish the two key treatments for testing the audience effect: territorial intrusion in the presence vs. in the absence of the audience. In fact, the only significant difference between treatments that was found was for *egr-1* expression in the Vs region between males in social isolation and males receiving a territorial intrusion in the absence of an audience. Thus, similarly to what has been recently described for zebrafish (Teles et al., 2015), our results do not support the functional localization hypothesis, since the different treatments could not be discriminated from the level of expression of *c-fos* or *egr-1* in any of the specific brain regions that have been sampled. In contrast to the lack of localized differences in the expression of any of the two immediate early genes used as reporters of neuronal activity, the patterns of co-expression of *c-fos* were specific for each treatment. This result supports the proposed hypothesis that audience effects are paralleled in the brain by a distributed processing of social information across the SDMN rather than in a specific brain region. Our results also suggest that the co-expression of *egr-1* across the SDMN nodes was more specific to the presence of an audience, since it only enabled the discrimination of the treatments with vs. without the presence of an audience, corroborating the analysis for *egr-1* expression in single brain regions of the SDMN discussed above.

Finally, the structural characterization of the state of the SDMN network as provided by network analysis of the patterns of co-expression of *c-fos* and *egr-1* across the nodes of the SDMN suggests an activation of the limbic system as given by the high centrality of Dm, Vv, and Vs in the *c-fos* network, and the higher centrality for these areas can be found in the treatments with territorial intrusions. The data for *egr-1* also suggests that the Vs is a central hub in all treatments. Moreover, the low centrality of the Vv and POA in the *c-fos* network of fish in social isolation can be linked to the absence of social interactions, since these areas have been identified as modulators of emotional stress reactivity and are also involved in sexual and aggressive behavior (O'Connell and Hofmann, 2011; Vindas et al., 2014). Our results also suggest that GC and TA are important nodes of the *c-fos* network. Although the TA is usually seen as an homolog of the mammalian ventromedial hypothalamus, there is evidence suggesting that only a subset of TA neurons may actually correspond to this brain region in mammals (Goodson and Kingsbury, 2013). Nevertheless, GC and TA are structurally connected (Kittelberger and Bass, 2013) and have been associated with vocal-acoustic behaviors (Goodson and Bass, 2002). It is unclear at this point if the connectivity to these areas is related to communication attempts with the audience, since although centrality measures for TA are higher when the audience is present with and without a territorial intrusion, it is also an important node for fish in social isolation.

In conclusion, our findings suggest that territorial males increase aggression and decrease androgen levels in response to territorial intrusions that take place in the presence of a female audience, and that these behavioral and hormonal responses are paralleled by changes in the pattern of activity of the brain SDMN, rather than by localized changes in a specific brain region.

## Author contribution

AR and RO designed the study; AR collected the data; AR, JL, and GO analyzed the data; AR and RO wrote the manuscript; all authors read and commented on the manuscript.

### Conflict of interest statement

The authors declare that the research was conducted in the absence of any commercial or financial relationships that could be construed as a potential conflict of interest.
