# Supplementary material for: Audience Effects in Territorial Defense of Male Cichlid Fish Are Associated with Differential Patterns of Activation of the Brain Social Decision-Making Network
Source: Front Behav Neurosci. 2017 May 31;11:105. doi: 10.3389/fnbeh.2017.00105 (PMC5449763; doi:10.3389/fnbeh.2017.00105)
Supplement: Supplementary file 1 [file Presentation1.PDF]

Supplementary material to
"Audience effects in territorial defense of male cichlid fish are associated with differential patterns of activation of the brain social decision-making network"

António Roleira[1], Gonçalo A. Oliveira[1,2], João S. Lopes[1,2] , Rui F. Oliveira[1,2,3*]
[1] ISPA – Instituto Universitário, Lisbon, Portugal
[2] Instituto Gulbenkian de Ciência, Oeiras, Portugal
[3] Champalimaud Foundation, Lisbon, Portugal
[*]Corresponding author: ruiol@ispa.pt

Table S1 - Primer sequence and RT-PCR parameters

**Gene**	**Accession Nº**	**Primer sequence (5' – 3')**	**Annealing temperature (Cº)**
Elf1a	HE608771.1	F – GGAGAGTTCGAGGCTGGTAT<br>R – TCTTGTTGACTCCGACGATGA	61
c-fos	XM_003455595.2	F – GACCTGTCCAGCTCCCTCTA<br>R – GCAGGTTACTACAGGTGTGC	59
egr-1	#AY493348.1	F– CCGTGGACACTCTGGGATA-3'<br>R – AAGGAGGCACTTGATGCTGT-3'	60

Detailed methods

Hormone assays

For cortisol assays we used the commercial antibody 'Anti-rabbit, Cortisol-3' [ref: 20-CR50, Interchim (Fitzgerald), Montluçon, France, cross-reactivity: cortisol 100%, prednisolone 36%, 11-Desoxycortisol 5.7%, Corticosterone 3.3%, Cortisone b 0.7%] and the radioactive marker [1,2,6,7-3H] Cortisol [ref: TRK407-250 mCi, Amersham Biosciences, NJ, USA]. Levels of free KT fraction were determined using an antibody kindly donated by D.E. Kime (see Kime and Manning, 1982, for cross-reactivity and other antibody details). The T antibody RDI was purchased from Research Diagnostics Inc. (Concord, USA) (ref: WLI-T3003, Rabbit anti-testosterone; cross reactivity: 5α-Dihydrotestosterone 63%; s-1-testosterone 46%; 5α-Androstan-3α,17β-diol 4.1%; s-5-Androsten-3β,17β-diol 14%; 5α-Androstan-3,17-dione < 4.0%; epi-testosterone < 0.7%; Aldosterone < 4.0%; Hydrocortisone, < 0.02%, Progesterone < 0.2%; Estradiol < 0.5%; Dehydroepiandrosterone< 0.4%; Androstenedione 3.5%; Danazol< 2.0%. The marked hormone for testosterone was the [1, 2, 6, 7-3H] Testosterone (Amersham Biosciences, ref. TRK402-250mCi).

Gene expression analysis

Total RNA was isolated from brain nuclei using the RNeasy Lipid Tissue Mini Kit with the following protocol:

RNA extraction

Tissue was homogenized in Qiazol lysis reagent and incubated for 7 min at room temperature (RT). Chloroform (1:2) was added, and the sample incubated at RT for 5 min. Samples were subsequently centrifuged at 13000 g for 20 min at 4oC, after which the upper aqueous phase was transferred to a new tube where 1 volume of 70% ethanol was added. This mixture was then transferred to an RNEasy column, remained 5 min at RT, and was centrifuged for 1 min at 9000 g. A sequence of buffers (provided by the RNeasy Lipid Tissue Mini Kit) was added to the Rneasy column: 700 µl of Buffer RW1, 500 µl of Buffer RPE and an additional 500 µl Buffer RPE. After each buffer, samples were centrifuged for 1 min at 9000 g and the flow-through was discarded. The RNeasy column was then placed in a new 2 ml tube and centrifuged for 3 min at 14000g. The column was transferred to a new 1.5 ml tube, RNA eluted with 25 µl of RNAse-free water, and centrifuged for 2 min at 9000 g. The elution step was repeated with the same 25 µl of RNAse-free water in order to increase RNA recovery efficiency. RNA concentration and purity of all samples was estimated by spectophotometric absorbance (260 nm and 280nm) in the Nanodrop (Thermo Scientific NanoDrop 2000), and the RNA integrity of a random group of samples was checked using Bionalyzer(Agilent 2100 Bioanalyzer).

Quantitative RT-PCR (qRT-PCR)

Primer sequences for qRT-PCR were designed on Primer 3 (Premier Biosoft International, Palo Alto, CA, USA), tested for quality in the FastPCR 5.4., and the PCR products were sequenced to confirm the amplicon (Table 1). qRT-PCR reactions were performed in an Applied Biosystems 7900HT Fast thermocycler in 8 µl triplicate reactions with SYBR Green PCR Master Mix (Applied Biosystems, Life Technologies) and primers at 50 µM. Thermocycling conditions were 5 min at 95º C, followed by 40 cycles of: 95º C for 30 s, specific annealing temperature for each primer for 30 s (Table 1), and 72º C for 30 s. After PCR, a melting curve program from 55 to 95º C with 0.5º C changes was applied and the presence of a single reaction product in each well was confirmed. All reactions were performed in triplicate and technical replicates were run on the same plate. Before the analysis, the threshold value was adjusted manually for each plate at the inflection point of the amplification curve, and the same threshold was used in all assays of the same gene.

Table S2 – Sample size by treatment for each variable

		NI.NA	NI.A	I.NA	I.A
Aggressive behavior	Bite Frequency	-	-	12	12
	Display Frequency	-	-	12	12
	Display Duration	-	-	12	12
Interactions with glass partition	Frequency	10	12	12	12
	Duration	10	12	12	12
Hormones	T	12	11	11	10
	11KT	12	12	9	11
	F	12	12	11	11
c-fos	Dm	10	10	8	8
	Dl	8	9	9	9
	Vv	10	7	6	7
	Vs	7	6	9	7
	POA	9	9	8	8
	TA	8	7	8	10
	GC	9	8	9	9
egr-1	Dm	12	11	10	10
	Dl	10	11	11	12
	Vv	10	9	7	9
	Vs	8	8	9	5
	POA	8	9	9	9
	TA	9	9	9	11
	GC	11	11	9	10

Table S3 – Number of outlier observations by treatment

		NI.NA	NI.A	I.NA	I.A
Interactions with the glass	Frequency	2	-	-	-
	Duration	2	-	-	-
Hormones	T	-	1	-	2
	KT	-	-	2	1
	F	-	-	-	1
c-fos	Vv	-	1	-	-
	Vs	1	-	-	-
	POA	-	-	-	1
	TA	-	-	1	-
egr-1	Vv	-	-	1	-
	POA	1	1	-	-
